# Seroprevalence of West Nile Virus in Blood Donors in Mainland Portugal

**DOI:** 10.3390/tropicalmed10080229

**Published:** 2025-08-15

**Authors:** Rafael Rocha, Elif Kurum, Rémi Charrel, Nazli Ayhan, Carla Maia

**Affiliations:** 1 Global Health and Tropical Medicine (GHTM), Associate Laboratory in Translation and Innovation Towards Global Health (LA-REAL), Instituto de Higiene e Medicina Tropical (IHMT), Universidade Nova de Lisboa (UNL), 1349-008 Lisboa, Portugal; rafael.amorim.rocha@gmail.com; 2 Unité des Virus Émergents (UVE), Aix-Marseille University, Università di Corsica, IRD 190, Inserm 1207, IRBA, 13005 Marseille, France; elif.kurum@etu.univ-amu.fr (E.K.); remi.charrel@univ-amu.fr (R.C.); nazli.ayhan@univ-amu.fr (N.A.); 3 National Reference Center for Arboviruses, Institut National de la Santé et de la Recherche Médicale (Inserm)-IRBA, 13005 Marseille, France

**Keywords:** flavivirus, West Nile virus, seroprevalence, blood donors, Portugal

## Abstract

The genus Orthoflavivirus includes several mosquito-borne pathogenic viruses, notably West Nile virus (WNV), which is endemic to the Mediterranean region. In Portugal, WNV circulation has been documented in equines, birds and mosquitoes, however human cases remain rare and no recent human seroprevalence studies have been conducted. This study aimed to estimate the national and regional seroprevalence of WNV among blood donors in mainland Portugal and explore associations with sociodemographic factors. A cross-sectional study conducted in 2022 included 3593 blood donors from across mainland Portugal. Serum samples were tested for WNV immunoglobulin G (IgG) by enzyme-linked immunosorbent assay (ELISA) and positive and borderline samples were confirmed using a virus neutralization test. Sociodemographic data were collected through a structured paper questionnaire. Statistical analyses, including multivariate logistic regression, identified factors associated with seropositivity. A total of 55 samples (1.5%) tested positive, and 21 samples (0.6%) were classified as borderline for WNV antibodies by ELISA. Of these, 47 were confirmed by viral neutralization, giving an estimated national seroprevalence of 1.4%. Significant regional variation was noted, with higher seroprevalence observed in the Beira Baixa, Grande Lisboa and Médio Tejo regions. Some seropositive individuals were identified in northern coastal regions such as Ave, Cávado and Área Metropolitana do Porto. In multivariate analysis, geographical area of residence was the only factor associated with seropositivity. This study highlights regions at potential higher risk for human WNV exposure, primarily in the southern half of Portugal. Continued and integrated surveillance is crucial to inform public health strategies to mitigate WNV transmission risks in these regions, as well as in other regions where WNV may be emerging as a relevant One Health concern. Implementing preventive measures for both animals and humans is critical to minimizing exposure and infection, and further confirmatory studies using virus neutralization tests will be important for refining these estimates.

## 1. Introduction

The genus *Orthoflavivirus*, belonging to the family Flaviviridae, comprises 53 species [1], several of which are pathogenic to humans and transmitted by mosquitoes such as Dengue virus (DENV), Zika virus (ZIKV) and Japanese encephalitis virus (JEV) [2]. Viruses of these species have garnered increasing attention from the global scientific community due to the high incidence rate and potential to cause epidemics [3], as well as the connection between their geographical spread and changes in the distribution of vectors, driven by climate change [4].

West Nile virus (WNV: *Orthoflavivirus nilense*) is maintained in an enzootic cycle, predominantly transmitted by Culex mosquitoes from avian reservoirs to host vertebrates. Birds are considered the primary reservoirs of WNV whereas equids, humans and other mammals are considered as dead-end hosts [5]. WNV is endemic in multiple European countries, particularly within the Mediterranean region [6], including in Portugal, where *Culex pipiens* appears to be the main vector [7]. Human cases of WNV disease are systematically monitored by the European Centre for Disease Prevention and Control (ECDC), along with surveillance in birds and equines by the European Food Safety Authority (EFSA). Surveillance data confirm the northward expansion of WNV, with recent detections in northern Germany and the Netherlands, as well as the continued transmission in the Iberian Peninsula, where annual cases have been reported in humans and equines in southwestern Spain since 2020 [8].

In humans, WNV infection is symptomatic in approximately 20–25% of cases, typically manifesting as a nonspecific, self-limiting febrile illness [9]. Less than 1% of infections result in neuroinvasive disease; however, these clinical forms (e.g., meningitis, encephalitis, acute flaccid paralysis) have a fatality rate of 10% and are associated with significant long-term morbidity [10]. A study of central nervous system (CNS) infections with unidentified agents after routine etiological investigation demonstrated that WNV might account for a substantial fraction (5.4%) of CNS infections in northern Italy [11]. Similar studies have suggested that WNV infection contributes to a significant percentage of suspected viral/aseptic meningoencephalitis cases (2.3–11.4%) in several other regions, including in Germany [12], Turkey [13], South Africa [14] and in the United States of America [15], especially in warmer months.

The epidemiological study of WNV infections has relied not only on the reporting of symptomatic cases in humans and other animals and virus detection in vectors, but also on cross-sectional serological studies of human populations in areas confirmed or potentially endemic for this virus. Some of these studies targeted blood donors in these regions. In the Mediterranean Basin, notable seroprevalence studies have been conducted in Italy, France and Spain, revealing seroprevalence rates of 0.08–0.6% in Spain [16,17,18], 0.8–1.2% in southern France [19,20] and 0–1.9% in Italy [21,22,23,24,25].

In Portugal, only one human seroprevalence study has been conducted to date, in the 1960s, showing a prevalence of 0.5%, by viral neutralization, among 1649 healthy individuals [26]. In addition, to date, no systematic screening of blood donors has been implemented in Portugal. Symptomatic human cases were diagnosed in 2004 (2 cases), 2010 (1 case) and 2015 (1 case), with the infection in these cases suspected to have been acquired in the NUTS2 (Nomenclature of Territorial Units for Statistics) regions of the Algarve and Península de Setúbal [27]. Additionally, the circulation of the virus between 1969 and 2022 has been demonstrated in equines, birds and mosquitoes in several parishes in the southern part of the country, particularly in the NUTS2 regions of the Algarve, Alentejo, Grande Lisboa and Península de Setúbal, and in a few isolated parishes in the northern half of the country [28]. Although only a limited number of WNV isolates from Portugal have been genetically characterized, available data indicate that lineage 1a is circulating in the country, having been identified in both birds [29] and mosquitoes [30] in previous studies.

However, *Culex pipiens* is widespread in Portugal, having been detected in almost every municipality in the country, between 2011 and 2022, according to national vector surveillance data [31]. Additionally, modeling studies using these data suggest that suitable habitats for WNV vectors are primarily located along the coast—including in the north of the country (Alto Minho, Área Metropolitana do Porto) and in the Área Metropolitana de Lisboa, as well as in the Tagus Valley—with precipitation identified as the main predictor; moreover, projections indicate that climate change will progressively expand ecological suitability for WNV transmission further north [27,28,32].

In this study, we aimed to estimate the national and regional seroprevalence of WNV in blood donors in mainland Portugal through the detection of antibodies using an enzyme-linked immunosorbent assay (ELISA) technique and to study the association between the presence of anti-WNV IgG antibodies and various sociodemographic factors in this population and the practices of individuals regarding occupation and daily activities.

## 2. Materials and Methods

### 2.1. Study Population

Samples and data for this study were originally collected as part of a separate cross-sectional *Leishmania* seroprevalence study [33]. A detailed description of the methodology is available in [33], with a summary provided in Supplementary Table S1. That study focused on the population of people who donate blood in mainland Portugal, recruited through the Portuguese Institute of Blood and Transplantation (IPST) and the Immunohemotherapy Departments (IHDs) of public hospitals in the Alentejo and Algarve regions. Both the IPST and IHDs conduct regular blood collections at fixed centers and mobile units operating in rural and urban areas. In 2021, these institutions oversaw over 190,000 blood donations [34], following a standardized triage protocol that includes clinical and epidemiological screening through a structured questionnaire and laboratory testing, in accordance with national and European Union blood safety regulations. Mainland Portugal, located in southwest Europe, bordering Spain and the Atlantic Ocean, is divided for statistical purposes into seven NUTS2 regions, 24 NUTS3 regions [35], 278 municipalities and 2882 parishes. According to the 2021 national census, the population of mainland Portugal aged 15 to 64 years was 6,257,752 [36].

### 2.2. Data and Sample Collection

The sampling process was stratified by municipality. Individuals participating in the original study attended one of the collaborating institutions between February and June 2022 and were deemed eligible for blood donation. Only individuals aged 18 to 65 years were included. Participant enrollment took place during non-randomly selected blood collection sessions, but within each session, invitations to participate were issued randomly, based on the time of arrival at the blood collection center or unit.

Participants completed a self-administered structured paper questionnaire addressing sociodemographic factors. From the routinely collected blood samples, 1.5 mL of serum was sent to the Instituto de Higiene e Medicina Tropical (IHMT) and stored at −20 °C for this study. Only those who had consented to participate in further studies were included in the WNV seroprevalence study.

Categorical variables extracted from the questionnaire were analyzed using the original categories provided as response options, with regrouping applied in certain cases. Profession classification followed the ESCO Classification of Occupations, developed by the European Commission since 2010 [37]. Parishes were classified as rural or non-rural based on the Portuguese Rural Development Program 2014–2020 criteria [38].

### 2.3. Serological Study

#### 2.3.1. Enzyme-Linked Immunosorbent Assay (ELISA)

Anti-WNV IgG antibody detection in each serum sample was performed using ELISA (WNV ELISA IgG, Euroimmun^®^, Lübeck, Germany) with EUROLabWorkstation automated system, following the manufacturer’s instructions and cut-offs. The ELISA used in this study has a reported sensitivity of 99.5% and specificity of 96.9%, according to the manufacturer. Each serum sample was tested once, with results categorized as positive, negative or borderline. Results are calculated semi-quantitatively by the ratio of extinction of the serum sample over the extinction of a calibrator. A signal was considered negative if the calculated ratio was <0.8, borderline if the ratio was ≤0.8–<1.1 and positive if it was ≥1.1.

#### 2.3.2. Virus Neutralization Test (VNT)

All ELISA positive and borderline samples and a sub-set of ELISA negative samples were tested using virus neutralization test with the WNV UVE/WNV/2023/FR/CNR_C1/G8 strain. This strain, belonging to lineage 2, was isolated from mosquitoes in France in 2023. Human sera were heat inactivated at 56 °C for 30 min, then dilutions from 1:10 to 1:80 were prepared by using the epMotion 5070 (Eppendorf) and mixed in 1:1 ratio with 100 TCID_50_ viral suspension of WNV (isolate Cx3-batch2_P0V_Zen-Pets-Res_2023-07-25) in 96-well plates. After a 1 h incubation, 100 μL of a Vero E6 cell suspension (5 × 10^5^ cells/mL) was added into the wells, and microplates were incubated at 37 °C in a 5% carbon dioxide incubator. After 5 days, the photos of the wells were taken by Incucyte SX5 (Sartorius^®^, Göttingen, Germany) and analyzed to identify the end dilution at which there was no cytopathic effect (CPE) and neutralization titers were determined. Detection of antibodies in any titer was considered as a positive result. The viral titer was calculated by using 50% tissue culture infectious dose (TCID50) by the Spearman–Kärber method [39].

### 2.4. Statistical Analysis

Crude prevalence was calculated for each NUTS2 and 3 region by dividing the number of positive samples by the total number of samples. To calculate the adjusted prevalence for each region, a correction was applied to the crude prevalence based on the population weight of each municipality or NUTS3 region within the corresponding NUTS3 or 2 region, respectively, considering the population aged 18 to 65 years.

Absolute and relative frequencies, along with hypothesis testing, were conducted using IBM^®^ SPSS^®^ Statistics Version 29.0. Geographical representation and analysis of the results were carried out using QGIS^®^ Version 3.40. Descriptive statistics were presented as absolute frequencies and percentages for categorical variables, and as medians with interquartile intervals (IQIs) for asymmetric continuous variables (e.g., age). Missing or unknown data were excluded from denominators unless otherwise specified. Group comparisons were performed using Pearson’s Chi-Square test for categorical variables, or Fisher’s exact test when Chi-Square assumptions were not met. Statistical significance was defined as *p* < 0.05. Confidence intervals (CI) for adjusted WNV seroprevalence were obtained using the Wilson’s method on Epitools© Epidemiological Calculators (Ausvet, 2025) [40,41].

A multivariate analysis was performed to identify sociodemographic factors associated with WNV infection. This was achieved through a multiple binary logistic regression model, including variables with statistical meaning in the univariate analysis (*p* < 0.20) as well as those deemed biologically relevant or potentially confounding.

For variables that remained significant in the model, crude odds ratios (OR) were updated to adjusted odds ratios (aOR) with 95% confidence intervals (CI). The goodness of fit for each logistic regression model was evaluated using the Hosmer–Lemeshow test [42]. The reference categories for each independent variable are detailed in the corresponding multivariate analysis results tables.

## 3. Results

A total of 3593 participants were included in this study, recruited across 636 blood collection sessions held at 347 different collection sites. Overall, 235 of the 278 municipalities in mainland Portugal were represented. The municipalities not represented were mainly located in the eastern Algarve, Alto Alentejo, Coimbra and Alto Minho NUTS3 regions. The median age of participants was 41 years, with higher median ages observed in the Península de Setúbal, Alentejo and Algarve NUTS2 regions (Table 1). Participants were evenly distributed between sexes overall and in most regions, except for the Alentejo and Algarve, where males were clearly predominant. Differences were also observed across NUTS2 regions regarding education level, occupation, contact with domestic animals and engagement in outdoor activities at night. These variations are detailed in Table 1.

### 3.1. Serological Results

**Figure 1:**
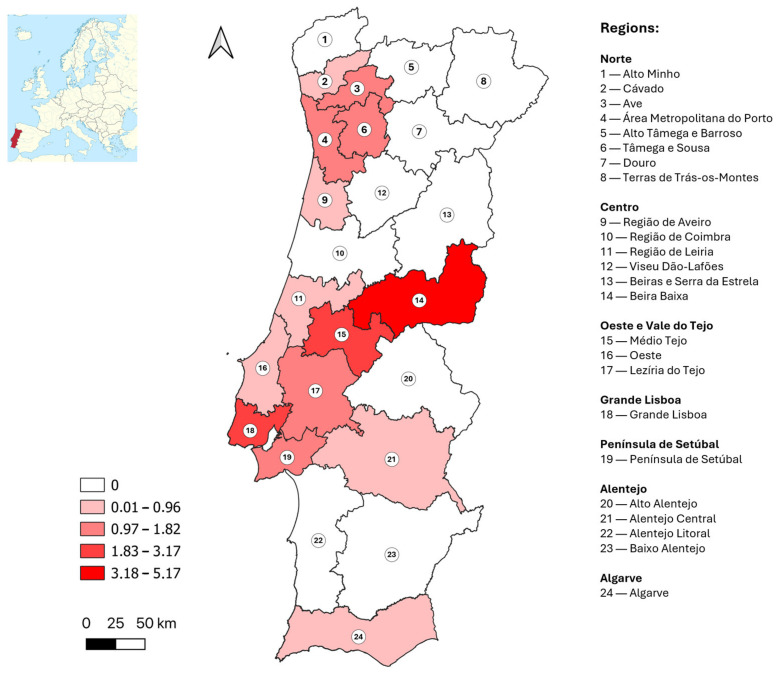
Distribution of estimated adjusted West Nile virus seroprevalence values (%) by NUTS (Nomenclature of Territorial Units for Statistics) 3 region (considering only viral neutralization test confirmed samples).

A total of 55 samples (1.5%) tested positive, and 21 samples (0.6%) were classified as borderline for WNV antibodies by ELISA. Of these 76 samples, 47 were considered positive by VNT (61.8%). The distribution of positive or borderline ELISA results and positive VNT results across NUTS2 and NUTS3 regions is presented in Table 2. Figure 1 illustrates the distribution of adjusted seroprevalence estimates by NUTS3 region, considering only VNT confirmed samples. Adjusted prevalences accounted for minor deviations between the expected and actual sample sizes by municipality.

The national adjusted seroprevalence estimate, considering only VNT confirmed samples, was 1.4% (95% CI: 1.0–1.8%). At the NUTS3 level, seroprevalence values ranged from 0.0% to 5.2%, with the highest rates observed in the Beira Baixa, Grande Lisboa and Médio Tejo regions. A statistically significant difference was found in the proportion of VNT positive results among the NUTS2 regions (*p* = 0.004, Chi-Square test, χ^2^ = 19.0, df = 6).

**Figure 2:**
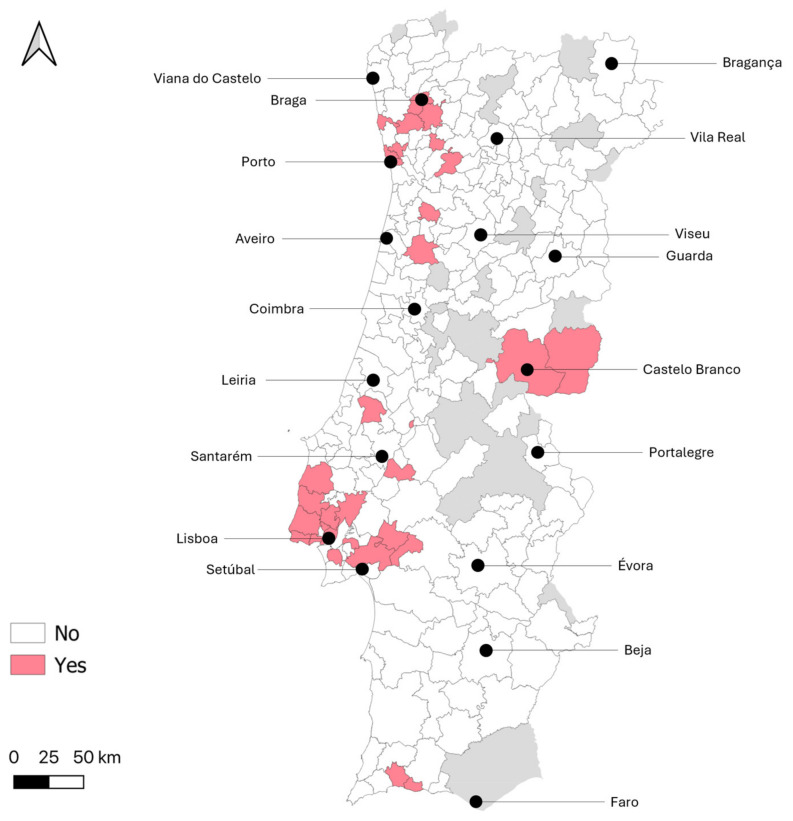
Geographic distribution of blood donors with positive West Nile virus serology (confirmed by viral neutralization test) by municipality of residence (the approximate location of district capital cities is highlighted; no data was collected for municipalities in grey).

To enable a more detailed analysis of the geographical distribution of potential exposure to WNV, the municipalities of residence of donors with WNV seropositive samples (confirmed by VNT) are displayed in Figure 2. Among the 73 municipalities where at least 15 samples were collected, those with the highest percentages of positive samples (>4.0%) were as follows, in descending order: Porto, Montijo, Póvoa de Varzim, Marco de Canaveses, Vila Franca de Xira and Lisboa.

### 3.2. Associations Between Sociodemographic Variables and Positive/Borderline Result

In the univariate analysis, factors associated with a positive WNV serological result (VNT confirmed) included the following: residing in the Grande Lisboa NUTS2 region (with Alentejo as the reference category); residing in a non-rural parish; and having no regular contact with domestic animals. Sex, age, practice of outdoor activities during nighttime and having traveled abroad within the past two years were not significantly associated with seropositivity. WNV positivity was higher in donors who worked in the armed forces and in people who reported having no mosquito nets in the windows/doors at home, although these differences did not reach statistical significance (Table 3).

In multivariate analysis, the only factor that remained significantly associated with a positive WNV serologic result (confirmed by VNT) was residing in the Grande Lisboa NUTS2 region (aOR 2.18, 95% CI 1.15–4.12, *p* = 0.017) (Table 4).

## 4. Discussion

This study provides an important step in understanding the epidemiology of WNV in mainland Portugal. By estimating the national and regional seroprevalence of WNV among blood donors and investigating associations between WNV seropositivity and sociodemographic factors, it offers a significant contribution to the existing knowledge on WNV in Europe, particularly in a country where systematic and recent human seroprevalence studies had not been previously conducted.

The study identified 1.3% of samples as positive (confirmed by VNT). This percentage was slightly higher than those observed in regional seroprevalence studies of blood donors in Spain, Italy and France, where most studies found rates below 1% [16,17,18,19,20,21,22,23,24,25]. In these studies, a similar serologic approach was used, where samples were initially tested by ELISA and ELISA-positive or borderline samples were confirmed using a second technique, often a neutralization test. While WNV ELISA demonstrates high sensitivity, its specificity is limited, partly due to cross-reactions with other flaviviruses [43], including those endemic to Europe, such as Usutu virus [44], as well as viruses that circulate extensively in tropical and subtropical regions, including yellow fever virus, dengue virus and Japanese encephalitis virus [45]. These cross-reactions can occur following natural infection with these viruses or after vaccination [46]. Previous vaccination against flaviviruses was not assessed in this study. Among flaviviruses, Usutu and Bagaza virus natural infection/exposure has recently been detected in Portugal in birds [47,48], with a prevalence in red-legged partridges (*Alectoris rufa*), in one of these studies, of 2.1 and 8.1%, respectively; however, as no country-wide studies in birds or seroprevalence studies in humans have been conducted, it is difficult to predict how the circulation of these viruses might have impacted our results. Neutralization tests are the most reliable serological assays, offering high specificity for differentiating among flaviviruses [49]. In our study, the use of VNT to confirm ELISA-positive or borderline results enhances the reliability of seroprevalence estimates, reducing the potential confounding effect of cross-reactivity with other flaviviruses; however, even with the VNT, cross-reactivity of antibodies between WNV and USUV should be considered when interpreting results, especially at low titers in areas where both viruses circulate.

Notably, a history of travel abroad within the last two years was not associated with higher WNV seroprevalence in our sample, suggesting limited influence from international exposure to flaviviruses. However, it is important to acknowledge that travel within Portugal or to other WNV endemic countries more than two years prior to sampling could have contributed to true WNV seropositivity reflecting exposure in locations other than participants’ current region of residence.

The study estimated an adjusted national WNV seroprevalence of 1.4% in Portugal (considering only VNT confirmed samples), with significant regional differences. Some of the regions with higher seroprevalence aligned with areas in the southern half of Portugal historically associated with WNV circulation (1969–2022) and projected to have higher ecological suitability for virus transmission [28]. These regions include Beira Baixa, Grande Lisboa, Médio Tejo, Península de Setúbal and Lezíria do Tejo. Conversely, no seropositive samples were identified in Alto Minho, Terras de Trás-os-Montes and Viseu Dão-Lafões, areas with no historical evidence of WNV circulation and predominantly low projected suitability [28]. Notably, some seropositive blood donors (without recent travel history) were identified in northern coastal regions, such as Ave, Cávado, Área Metropolitana do Porto and Região de Aveiro, where WNV detection has been sporadic or absent. Models, however, predict that certain areas of Região de Aveiro currently have high suitability, and this suitability may be expanding further north due to climate change [28]. Moreover, *Culex pipiens* is widely distributed in these regions [31], and some of the most favorable habitats for this species in Portugal seem to be located along these coastal areas [32].

In Portugal, WNV fever is a mandatory notifiable disease in equids [50]. Since 2015, outbreaks in horses have been reported annually. In recent years, the number of outbreaks has increased, rising from one in 2018 to 17 in 2024 [51]. The regions with the highest number of outbreaks were, in descending order, Lezíria do Tejo, Alentejo Central and Algarve [51]; seropositive individuals were detected in all of these regions in the present study. Furthermore, among the 20 municipalities where equid outbreaks were detected between 2015 and 2024 and were sampled in this study, three had blood donors with positive VNT results (namely Castelo Branco, Lagoa and Vendas Novas), highlighting some overlap in the findings. The finding of seropositive donors in several municipalities in coastal northern Portugal could suggest ongoing undetected transmission in this area and reinforces the need to intensify surveillance efforts in horses nationwide. Although testing and reporting in other mammals are not consistently conducted in Portugal or Spain, species that have tested positive for WNV antibodies, such as dogs and lagomorphs, have been suggested as potential additional sentinels for monitoring WNV in Iberian Mediterranean ecosystems [52,53], along with wild birds [54].

Few studies in Europe have explored the associations between sociodemographic factors and human (asymptomatic) seropositivity for WNV, likely due to the low number of seropositive individuals in these studies. In those that conducted multivariate analyses, sex and age were not significantly associated with a positive result, which aligns with the findings of the present study [18,22,25]. Interestingly, multiple studies in symptomatic patients have suggested an association between male sex, older age and an increased risk of WNV fever and neuroinvasive disease [55,56].

One seroprevalence study [18] did find that a risk profession (involving close contact with animals, nature, wetlands, or mosquitoes) was associated with seropositivity in univariate analysis. In the present study, blood donors working in the armed forces had a higher seropositivity for WNV, which could be related to increased exposure during outdoor activities, especially in areas where vectors are common, such as wetlands. However, this difference was not statistically significant. Due to data limitations, a more detailed analysis of other professions or an indoor versus outdoor occupational classification was not feasible.

The evidence of active WNV circulation in Portugal highlights the need for robust surveillance systems, including vector monitoring, seroprevalence studies and real-time reporting of human and animal cases. Such systems can provide early warnings of outbreaks and facilitate timely interventions. Integrated strategies, such as the elimination of mosquito breeding sites and the use of insecticides, should prioritize high-prevalence areas, where there is potentially greater WNV exposure, such as Grande Lisboa.

Raising awareness about WNV transmission and prevention among at-risk populations, including military personnel, can reduce exposure. Educational campaigns should emphasize the use of protective measures, such as insect repellent and appropriate clothing, particularly during peak mosquito activity periods. Raising awareness among physicians is also crucial, as it can lead to increased recognition and timely reporting of cases. Encouraging routine consideration of WNV in differential diagnoses of febrile syndromes and CNS infections in warmer months, particularly in regions where recent circulation of the virus has been documented, can help identify cases that might otherwise go unrecognized. However, diagnostic capacity for WNV infection remains limited in many clinical laboratories in Portugal, and testing, including confirmatory assays, often relies on referral to specialized or reference laboratories [57], which can delay case identification and response. Lastly, the detection of seropositive healthy people in some regions in Portugal, suggesting ongoing vectorial transmission, raises concerns about the potential for transfusion-transmitted WNV infections in these regions, especially in the southern half of Portugal. Following a symptomatic human case in the Algarve in 2015, screening was temporarily implemented in the region, using nucleic acid amplification tests (NAAT), but no positive donors were identified thereafter. However, given the consistent observation of WNV circulation in this and other areas of the country, broader and sustained screening measures could be considered, including the use of NAAT during peak transmission seasons. Continuous surveillance remains crucial to inform and optimize blood donation screening practices.

This study has several strengths, including its robust sample size of 3593 participants, calculated (for the original study) to ensure adequate national and regional precision of seroprevalence estimation. Additional strengths include the extensive geographical representation across 196 municipalities of mainland Portugal and the use of the gold standard serologic technique to confirm ELISA positive or borderline results. Additionally, the inclusion of a wide range of sociodemographic and behavioral variables allows for a comprehensive analysis of potential risk factors.

However, there are notable limitations. First, the study population consisted exclusively of blood donors, who may not be representative of the general population due to selection bias. Blood donors are typically healthy adults, potentially underestimating seroprevalence in vulnerable groups, such as the elderly or those with underlying health conditions. In addition, the representativeness even of the blood donor population itself could have been affected by the difficulty in obtaining a truly probabilistic sample, due to logistic constraints in some regions.

Despite the many caveats for the use of serology for individual determination of previous WNV infection status, the detection of antibodies can be relevant from a public health perspective, especially when comparing the findings between different regions and by crossing the results with the distribution of human cases and the evidence from WNV in mammals, birds and vectors, following a One Health approach.

## 5. Conclusions

This study represents a pivotal step in understanding WNV epidemiology in mainland Portugal, providing valuable insights into its national and regional seroprevalence and associated risk factors. The study reinforces WNV activity in previously known areas (such as Península de Setúbal and the Algarve) and suggests its likely circulation in areas not previously documented (including northern coastal regions). While the study’s design focused on blood donors, the results underscore the importance of raising clinical awareness of WNV, particularly in regions with higher seroprevalence and during the warmer season. Strengthening diagnostic infrastructure and ensuring broader access to reliable WNV testing is also essential to translate increased clinical suspicion into effective detection. These findings also highlight the need for targeted and integrated surveillance, following a One Health approach, to inform public health interventions to mitigate the impact of WNV in Portugal, particularly in light of ongoing climate change and its influence on vector-borne disease dynamics.

## Figures and Tables

**Table 1:** Sociodemographic characteristics of the participants, globally and by NUTS (Nomenclature of Territorial Units for Statistics) 2 region.

	Global	Norte	Centro	OVT	GL	PS	Alentejo	Algarve
Total	100	33.6	14.7	8.1	19.4	7.6	10.0	6.6
	(3572/3572)	(1201/3572)	(527/3572)	(289/3572)	(692/3572)	(270/3572)	(358/3572)	(235/3572)
Median age (years)	41	39	40	41	41	44	43	42
(IQR)	(31–48)	(30–47)	(29–47)	(29–49)	(30–50)	(34–49)	(35–50)	(35–50)
Male sex (%)	49.8	47.3	47.1	50.9	48.7	44.8	61.7	57.5
	(1774/3564)	(567/1198)	(248/526)	(147/289)	(336/690)	(121/270)	(221/358)	(134/233)
Education level ^a^								
Basic (1–4)	1.7	1.6	1,4	3.1	1.0	1.1	3.4	1.7
	(60/3483)	(18/1161)	(7/514)	(9/286)	(7/677)	(3/265)	(12/350)	(4/230)
Basic (5–9)	16.6	20.0	16.3	18.2	9.6	14.0	19.7	17.0
	(578/3483)	(232/1161)	(84/514)	(52/286)	(65/677)	(37/265)	(69/350)	(39/230)
Secondary (10–12)	44.0	43.2	42.4	49.3	39.6	39.6	51.1	51.3
	(1531/3483)	(502/1161)	(218/514)	(141/286)	(268/677)	(105/265)	(179/350)	(118/230)
Bachelor’s	26.0	23.6	28.8	22.7	31.9	32.5	20.0	20.9
	(907/3483)	(274/1161)	(148/514)	(65/286)	(216/677)	(86/265)	(70/350)	(48/230)
MSc/PhD	11.7	11.6	11.1	6.6	17.9	12.8	5.7	9.1
	(407/3483)	(135/1161)	(57/514)	(19/286)	(121/677)	(34/265)	(20/350)	(21/230)
Occupation ^b^								
Student	9.9	9.3	13.6	12.4	12.6	5.5	5.7	4.3
	(283/2866)	(89/960)	(59/435)	(30/241)	(69/546)	(12/219)	(16/279)	(8/186)
Unemployed	3.5	4.5	2.8	3.3	3.7	2.7	1.4	3.2
	(99/2866)	(43/960)	(12/435)	(8/241)	(20/546)	(6/219)	(4/279)	(6/186)
Retired	1.7	1.0	0.7	1.7	2.9	1.4	3.2	2.7
	(50/2866)	(10/960)	(3/435)	(4/241)	(16/546)	(3/219)	(9/279)	(5/186)
Armed forces (0)	1.9	1.4	1.6	3.3	1.1	2.7	2.5	3.8
	(54/2866)	(13/960)	(7/435)	(8/241)	(6/546)	(6/219)	(7/279)	(7/186)
Managers, professionals and technicians (1–3)	39.4	37.2	37.7	31.5	50.0	49.3	33.0	31.2
	(1128/2866)	(357/960)	(164/435)	(76/241)	(273/546)	(108/219)	(92/279)	(58/186)
Clerical support, service and sales (4–5)	25.7	24.3	22.5	26.1	20.0	24.2	35.1	44.6
	(737/2866)	(233/960)	(98/435)	(63/241)	(109/546)	(53/219)	(98/279)	(83/186)
Agriculture, craft, industry and elementary (6–9)	18.0	22.4	21.1	21.6	9.7	14.2	19.0	10.2
	(515/2866)	(215/960)	(92/435)	(52/241)	(53/546)	(31/219)	(53/279)	(19/186)
Others								
Regular contact with domestic animals	70.8	70.5	79.4	74.9	61.4	68.6	74.3	73.3
	(2410/3404)	(799/1134)	(397/500)	(209/279)	(409/666)	(179/261)	(252/339)	(165/225)
Practice of outdoor activities during nighttime	24.6	19.9	29.2	26.9	23.2	23.4	33.0	27.6
	(798/3248)	(217/1088)	(138/473)	(71/264)	(148/637)	(59/252)	(107/324)	(58/210)

Abbreviations: GL—Grande Lisboa; IQR—Interquartile range; MSc—Master of Science; OVT—Oeste e Vale do Tejo; PhD—Doctor of Philosophy; PS—Península de Setúbal; (a) Numbers in brackets refer to number of years completed of formal school education; (b) Numbers in brackets refer to the numbers of the categories in the classification of European Skills, Competences and Occupations.

**Table 2:** Distribution of positive or borderline results by enzyme-linked immunosorbent assay and positive results by viral neutralization test, by NUTS (Nomenclature of Territorial Units for Statistics) 2 and 3 regions and estimated crude and adjusted West Nile virus prevalence.

Region	Sampling Sites (*n*)	Total Samples (*n*)	Positive or Borderline Samples, ELISA (*n*)	Positive Samples, VNT (*n*)	Crude Prevalence, VNT (%)	Adjusted Prevalence, VNT (%)	95% Confidence Interval
Norte	149	1201	17	13	1.1	1.1	0.6–1.8
Alto Minho	12	68	0	0	0.0	0.0	0.0–5.3
Cávado	17	148	3	1	0.7	0.8	0.1–3.7
Ave	16	143	2	2	1.4	1.4	0.4–5.0
Área Metropolitana do Porto	60	555	10	8	1.4	1.5	0.7–2.8
Alto Tâmega e Barroso	4	29	0	0	0.0	0.0	0.0–11.7
Tâmega e Sousa	23	151	2	2	1.3	1.4	0.4–4.8
Douro	13	75	0	0	0.0	0.0	0.0–4.9
Terras de Trás-os-Montes	4	32	0	0	0.0	0.0	0.0–10.7
Centro	91	527	7	4	0.8	0.6	0.2–1.7
Região de Aveiro	19	134	2	1	0.7	0.9	0.1–4.2
Região de Coimbra	21	108	1	0	0.0	0.0	0.0–3.4
Região de Leiria	14	99	1	1	1.0	0.9	0.2–5.1
Viseu Dão-Lafões	15	65	0	0	0.0	0.0	0.0–5.6
Beira Baixa	7	40	2	2	5.0	5.2	1.4–16.5
Beiras e Serra da Estrela	15	81	1	0	0.0	0.0	0.0–4.5
Oeste e Vale do Tejo	38	289	5	3	1.0	1.1	0.4–3.0
Oeste	12	139	1	1	0.7	0.9	0.1–4.2
Médio Tejo	16	64	2	1	1.6	2.2	0.3–8.9
Lezíria do Tejo	10	86	2	1	1.2	1.2	0.2–6.3
Grande Lisboa	16	692	28	20	2.9	3.0	2.0–4.6
Península de Setúbal	9	270	7	4	1.5	1.4	0.6–3.6
Alentejo	42	358	5	1	0.3	0.2	0.0–1.6
Alentejo Litoral	10	69	0	0	0.0	0.0	0.0–5.3
Baixo Alentejo	8	100	1	0	0.0	0.0	0.0–3.7
Alto Alentejo	9	55	0	0	0.0	0.0	0.0–6.5
Alentejo Central	15	134	4	1	0.7	0.7	0.1–4.1
Algarve	2	235	7	2	0.9	0.7	0.2–2.8
Total	347	3572	76	47	1.3	1.4	1.0–1.8

Abbreviations: ELISA—enzyme-linked immunosorbent assay; *n*—number; VNT—viral neutralization test.

**Table 3:** Distribution of participants by serological result and by category, for sociodemographic variables.

		Result of WNV Serology		
Variables	Categories	ELISA and VNT Positive	ELISA Negative	*p* Value
Sex	Male	51.1 (24/47)	49.7 (1734/3488)	0.854 (χ^2^ = 0.34, df = 1)
	Female	48.9 (23/47)	50.3 (1754/3488)	
Age (years)	18–24	10.6 (5/47)	12.9 (442/3413)	0.381 (χ^2^ = 4.19, df = 4)
	25–34	27.7 (13/47)	20.2 (688/3413)	
	35–44	21.3 (10/47)	28.7 (979/3413)	
	45–54	34.0 (16/47)	27.0 (921/3413)	
	55–65	6.4 (3/47)	11.2 (383/3413)	
Level of education ^a^	1–4	0.0 (0/47)	1.7 (59/3407)	0.125 (FET = 6.79)
	5–9	6.4 (3/47)	16.8 (573/3407)	
	10–12	46.8 (22/47)	43.9 (1495/3407)	
	Bachelor’s	25.5 (12/47)	25.9 (884/3407)	
	MSc/PhD	21.3 (10/47)	11.6 (396/3407)	
Occupation ^b^	Student	7.7 (5/65)	9.9 (278/2801)	
	Retired	4.6 (3/65)	1.7 (47/2801)	
	Unemployed	3.1 (2/65)	3.5 (97/2801)	
	0	8.8 (3/34)	2.1 (49/2379)	0.066 (FET = 6.81)
	1–3	52.9 (18/34)	46.2 (1100/2379)	
	4–5	20.6 (7/34)	30.4 (724/2379)	
	6–9	17.6 (6/34)	21.3 (506/2379)	
Travel abroad (<2 years previously)	Yes	29.8 (14/47)	23.7 (815/3437)	0.331 (χ^2^ = 0.94, df = 1)
	No	70.2 (33/47)	76.3 (2622/3437)	
Type of parish of residence	Non-rural	76.6 (36/47)	57.6 (2012/3492)	0.009 * (χ^2^ = 6.85, df = 1)
	Rural	23.4 (11/47)	42.4 (1480/3492)	
Regular contact with domestic animals	Yes	51.1 (23/45)	71.0 (2366/3331)	0.004 * (χ^2^ = 8.52, df = 1)
	No	48.9 (22/45)	29.0 (965/3331)	
Regular contact with wild animals	Yes	4.7 (2/43)	4.1 (131/3159)	0.699 (FET = 0.50)
	No	95.3 (41/43)	95.9 (3028/3159)	
Practice of outdoor activities during nighttime	Yes	22.7 (10/44)	24.4 (777/3178)	0.792 (χ^2^ = 0.07, df = 1)
	No	77.3 (34/44)	75.6 (2401/3178)	
Use of nets in windows/doors	Yes (all/some)	13.3 (6/45)	24.0 (799/3331)	0.096 (χ^2^ = 2.78, df = 1)
	None	86.7 (39/45)	76.0 (2532/3331)	
NUTS2 region of residence	Norte	27.7 (13/47)	33.9 (1184/3496)	0.011 * (FET = 15.36)
	Centro	8.5 (4/47)	14.9 (520/3496)	
	Oeste e Vale do Tejo	6.4 (4/47)	8.1 (284/3496)	
	Grande Lisboa	42.6 (20/47)	19.0 (664/3496)	
	Península de Setúbal	8.5 (4/47)	7.5 (263/3496)	
	Alentejo	2.1 (1/47)	10.1 (353/3496)	
	Algarve	4.3 (2/47)	6.5 (228/3496)	

Abbreviations: ELISA—enzyme-linked immunosorbent assay; FET—Fisher’s Exact Test; MSc—Master of Science; NUTS—Nomenclature of Territorial Units for Statistics; PhD—Doctor of Philosophy; VNT—viral neutralization test; WNV—West Nile virus; ^a^ Categories refer to the number of years completed of formal school education; ^b^ Category numbers refer to the numbers of the categories in the classification of European Skills, Competences and Occupations; * Statistically significant.

**Table 4:** Potential risk factors for West Nile virus infection, according to logistic regression models to estimate crude and adjusted odds ratio values.

Potential Risk Factor	Univariate			Multivariate		
	% in Sample	Crude OR	95% CI	Adjusted OR	95% CI	*p*-Value
Age ≥ 45 years old	38.3	1.10	0.61–1.97	1.04	0.56–1.93	0.607
Male sex	49.8	1.06	0.59–1.88	1.00	0.55–1.83	0.953
Residing in Grande Lisboa region	19.4	3.16	1.76–5.67	2.18	1.15–4.12	0.017 *
Residing in a non-rural parish	57.9	2.41	1.22–4.74	1.37	0.64–2.93	0.672
Higher education level	37.7	1.46	0.82–2.60	1.28	0.69–2.38	0.826
No use of nets in windows/doors	76.1	2.05	0.87–4.86	1.72	0.72–4.12	0.815
No regular contact with domestic animals	29.2	2.35	1.30–4.23	1.53	0.74–3.16	0.310
Constant				0.018		<0.001
Hosmer and Lemeshow Test				Sig. = 0.945		

Abbreviations: CI—confidence interval; OR—odds ratio; * Statistically significant.

## Data Availability

The datasets generated and analyzed during the current study are not publicly available due to confidentiality commitment with the participants, as stated in the consent declaration, but are available from the corresponding author on reasonable request.

## Supplementary Materials

The following supporting information can be downloaded at: https://www.mdpi.com/article/10.3390/tropicalmed10080229/s1. Table S1: Summary of the methodology of the previous study—cross-sectional study on the prevalence of anti-*Leishmania* antibodies in blood donors in mainland Portugal. References [58,59,60,61,62] are cited in the supplementary materials.
